# Viscossuplementation for the treatment of osteoarthritis of the knee: A protocol for an umbrella review of systematic reviews with meta-analyses of randomized controlled trials

**DOI:** 10.1097/MD.0000000000021813

**Published:** 2020-09-11

**Authors:** Andrade Carlos Augusto Ferreira de, Isabel Ruguê Genov, Sara Regina Neto Pereira, Joao Mauricio Barreto, Max Rogério Freitas Ramos, Eduardo Costa Freitas da Silva, Liszt Palmeira de Oliveira

**Affiliations:** a Unimed-Rio Institute - Rio de Janeiro; b Department of Epidemiology and Quantitative Methods in Health, National School of Public Health Sergio Arouca (ENSP)/Oswaldo Cruz Foundation (Fiocruz); c Faculty of Medicine - Vassouras University; d Nove de Julho University, São Paulo; e Faculty of Medical Sciences, State University of Rio de Janeiro; f National Institute of Traumatology and Orthopedics Jamil Haddad - Rio de Janeiro; g Orthopaedic Department, Federal University of Rio de Janeiro - Rio de Janeiro; h Department of Internal Medicine; i Department of Surgical Specialties, State University of Rio de Janeiro, Rio de Janeiro, Brazil.

**Keywords:** knee osteoarthritis, umbrella review, viscosupplementation

## Abstract

**Background:**

Knee osteoarthritis (KOA) is a common chronic disease with worldwide prevalence of 10% to 79%, with costs ranging from $560 to $635 billion for year in United States of America. The main guidelines recommend interventions with undesirable adverse events (AE) or highly dependent on the patient's persistence. Thus, intra-articular (IA) therapies appear to be attractive in patients with KOA, as well as a valid therapy by maximizing effects locally in the joint and limiting systemic AE. Presently, the main available IA therapies are corticosteroids and hyaluronic acid.

As several meta-analyses about the efficacy of intra-articular hyaluronic acid (IAHA) for treatment of KOA with discordant results were published, we decided to conduct an umbrella review to summarize this efficacy

**Methods:**

We will search MEDLINE/PubMed, EMBASE, Cochrane Library, and Virtual Health Library (BVS) from inception to February 2020 for systematic reviews with meta-analyses of randomized clinical trials that investigate IAHA for therapy of KOA. Grey literature will be searched in Opengray platform, Research Gate, and Google Scholar. The reference lists of eligible studies will be screened. The search will be performed without language restriction.

We will include any type of IAHA as experimental intervention and different types of oral or intra-articular placebo or medications as controls. The primary outcome will be measures of efficacy as the Western Ontario and McMaster Universities Osteoarthritis Index.

A synthesis of the evidence will be conducted and data will be presented in tables.

Two reviewers will independently appraise the quality of included meta-analyses using the Assessment of Multiple Systematic Reviews 2 (AMSTAR 2) tool and will classify the included systematic reviews into high, moderate, low, or critically low levels of confidence.

**Results:**

The results of this study will be published in a peer-reviewed journal.

**Ethics and dissemination:**

No ethical approval is required since this study data is based on published literature.

**Protocol registration number:**

PROSPERO CRD42019120269 (https://www.crd.york.ac.uk/PROSPERO/#joinuppage).

## 1 Introduction

Osteoarthritis (OA) is one of the most prevalent chronic diseases.^[1]^ Clinically, the knee is the most common site of osteoarthritis accounting for approximately 85% of the burden of osteoarthritis.^[1]^ The worldwide prevalence of symptomatic knee osteoarthritis (KOA) ranges from 9% to 38% for women and from 3% to 14% for men.^[2]^ These numbers are more striking if we consider the prevalence of radiographic KOA: 10% to 79% for women and 10% to 58% for men.^[2]^

Considering the aging of the world population, as KOA is a frequent condition in the elderly it appears that in 2010 it affected 3.64% people around the world.^[3]^ A Swedish registry data study estimated, by 2032, an increase of the proportion of people aged 45 years and older with doctor-diagnosed knee osteoarthritis from 13.8% to 15.7%.^[4]^

Two studies^[5,6]^ showed that the effects of age on risk of KOA in women is increasing, with peaks in incidence around the age of 75 years. This increase is attributable to the global ageing population and obesity epidemic.^[1]^

Considering all the sites involved in OA, this disease was considered the sixth cause of the Level 3 causes of global age-specific years lived with disability in 2015 for the age groups of 60 to 64 years and 65 to 69 years.^[1]^ Knee osteoarthritis accounts for approximately 85% of the burden of OA worldwide.^[1]^ This condition causes a negative mental and physical impact on these patients.^[7,8]^ Elderly people are the most affected by this disease, suffering with pain, functional limitation, and impairment of life quality.^[1,9]^ It was shown in the literature^[9]^ that KOA affects the whole body, affecting the patients’ lives on several ways.

So, we see that OA, mainly KOA, is a very important cause of disability and its responsible for a considerable burden on the individuals affected.^[7,8]^ Consequently, KOA's direct and indirect costs are expected to be very high. Estimates from 2009 showed that KOA affects almost 9 million adults in the United States of America and accounts for about $27 billion in the costs annual health care.^[10]^ If we include lost productivity the costs of KOA increases and ranges from $560 to $635 billion.^[11,12]^

Since KOA is predominantly a degenerative disease, despite having an inflammatory component, it can be assumed that there is great difficulty in finding an effective non-surgical treatment for this condition.^[2]^ Thus, even with the recent advances in knowledge of KOA pathophysiology, as well as the development of new molecules, to date there are no medications or surgical interventions that demonstrated to alter the course of KOA development.^[13]^

Consequently, taking into account the aspects presented above, currently the treatment of KOA ultimate goal is to relieve symptoms and to improve joint function and resulting in an improvement in the quality of life of patients.^[14]^

Possibly, total knee replacement (TKA) is an effective and adequate treatment for patients with advanced stages of KOA, but the risk of complications of this procedure presents risks, especially if we consider that most patients with KOA have advanced age and/or many comorbidities.^[14]^

The main guidelines for KOA management^[13,15–17]^ recommend conservative interventions such as oral and topical nonsteroidal anti-inflammatory drugs (NSAID's), weight loss, strength training, water and land-based exercise, self-management skills as the first line of treatment. Notwithstanding, NSAIDs have many undesirable drug-related adverse events (AEs), mainly in elderly patients who are most affected by the disease.^[18]^ On the other hand, however, as this type of treatment is highly dependent on the patient's persistence, there is low adherence to non-pharmacological and non-surgical therapies.^[19]^ In addition, most non-pharmacological and non-surgical treatments (e.g., diets for weight reduction, and physical exercises) need to be performed frequently and constantly. If there is interruption of treatment and/or if this kind of therapy is carried out with “low intensity,” its effectiveness will be greatly compromised.

Thus, intra-articular (IA) injection therapies appear to be an attractive alternative in patients diagnosed with KOA, as well as a valid adjunctive therapy by maximizing therapeutic effects locally in the joint and limiting potential systemic adverse effects.^[8,20]^ Presently, the available intra-articular therapies are corticosteroids, viscosupplements (hyaluronic acid) and, in some countries, agents such as blood-derived products (platelet-rich plasma).^[21,13–15,17]^ Recently published clinical guidelines^[13]^ indicate the use of intra-articular corticosteroids (IACS) and hyaluronic acid (IAHA) for KOA as recommendations (Level 1B—75% of the panel members who drafted the recommendations in favor and >50% conditional recommendation and Level 2—60–74% in favor). These guidelines^[13]^ highlight that considering pain as outcome, IAHA may have profitable effects at and beyond 12 weeks of therapy, and also, a more favorable long-term safety profile than repeated IACS. However, if the outcome was knee function, both IACS and IAHA have showed similar efficacy.^[8]^

Although there is evidence of the safety of IAHA for multiple courses of injection,^[22]^ the meta-analyses that assessed the efficacy of this intervention found discordant results.^[23]^ Besides that, it was shown that IAHA was the most effective treatment for KOA pain in a network meta-analysis which analyzed 137 studies comprising 33,243 patients.^[24]^

As several meta-analyses about the efficacy of IAHA for treatment of KOA with discordant results were published and we found the record of only 1 umbrella review protocol about a similar subject (our review is specific for KOA, the other is about viscosupplementation for osteoarthritis in general, with no specific site^[25]^ with the anticipated completion date was August 1, 2017, and we know that it will not be published soon^[26]^), we decided to conduct an umbrella systematic review to summarize the efficacy of IAHA for treatment of KOA and assess the quality of the meta-analyzes already published on this important topic.

## 2 Methods

### 2.1 Study registration

As there is no specific guideline for reporting umbrella reviews protocols, we decided to use the preferred reporting items for systematic review and meta-analysis protocols (PRISMA-P)^[27]^ guideline and this protocol was prepared according to this guideline.

The protocol scheme matches the PRISMA-P^[27]^ reporting standards. The study protocol has been registered on International prospective register of systematic reviews (PROSPERO) (https://www.crd.york.ac.uk/prospero) with a unique ID of CRD42019120269.

### 2.2 Eligibility criteria

#### 2.2.1 Type of study

We will include systematic reviews with meta-analyses of randomized clinical trials that investigate the safety and the efficacy/effectivity of IAHA for therapy of KOA. Systematic reviews of randomized clinical trials without meta-analysis and meta-analyses without systematic reviews will be rejected.

#### 2.2.2 Participants

We will include patients with KOA. No limitations of clinical features, age, race, nation, or sex will be set on the participant's characteristics.

#### 2.2.3 Interventions and controls

We will include any type of IAHA as experimental intervention. We will include different types of oral or intra-articular placebo, intra-articular corticosteroids, NSAIDs (topical or oral), and other pharmacological agents placebo, sham procedures, active control, or no treatments as controls.

#### 2.2.4 Outcome assessments

The primary outcome will be measures of efficacy as the visual analog scale (VAS) for pain, the Western Ontario and McMaster Universities Osteoarthritis Index (WOMAC) for pain, function, and/or stiffness, the Lequesne Index, the Brief Pain Inventory; the Knee Injury and Osteoarthritis Outcomes Score (KOOS), and the Animated Activity Questionnaire.^[13]^

### 2.3 Data source and search strategy

A systematic search of the available manuscripts will be conducted in electronic databases MEDLINE/PubMed, EMBASE, Cochrane Library, and Virtual Health Library (BVS) from inception to February 2020 for systematic reviews with meta-analyses of randomized clinical trials that investigate the safety and the efficacy/effectivity of IAHA for therapy of KOA. Grey literature will be searched in Opengray platform, Research Gate, and Google Scholar. In addition, the reference lists of potentially eligible studies will also be screened in order to verify all relevant items not identified during searches in databases (cross references). Regarding the language, there will be norestriction on the publication of the language of articles. No initial publication date limit will be set.

The following Medical Subject Headings (MeSH) items, free words or publication type will be taken: “meta analyses,” “meta analysis,” “injections, intraarticular,” injections, “intra articular,” “hyaluronic acid/therapeutic use,” “Knee, Osteoarthritis Of,” “viscosupplementation,” “osteoarthritis, knee,” “osteoarthritides,” “knee osteoarthritis,” “osteoarthritis of knee,” and “osteoarthritis of knees,” generating the following strategy for MEDLINE /PubMed: (((((“meta analyses”[Text Word]) OR “meta analysis”[Text Word]) OR “meta analysis”[Publication Type])) AND (((((“injections, intraarticular”[MeSH Terms]) OR “injections, intra articular”[Text Word]) OR “hyaluronic acid/therapeutic use”[MeSH Terms]) OR “viscosupplementation”[MeSH Terms]) OR “viscosupplementation”[Text Word])) AND ((((((Knee, Osteoarthritis Of[Text Word]) OR “osteoarthritis, knee”[MeSH Terms]) OR “osteoarthritides”[Text Word]) OR “knee osteoarthritis”[Text Word]) OR “osteoarthritis of knee”[Text Word]) OR “osteoarthritis of knees"). We will use equivalent strategies in the other databases.

We will also search the websites of PROSPERO, Osteoarthritis Research Society International (OARSI), European League Against Rheumatism (EULAR), American College of Rheumatology (ACR), repositories of theses and dissertations, and we will contact experts opinions for relevant systematic reviews with meta-analyses.

### 2.4 Study selection

Studies that meet the aforementioned eligibility criteria will be considered for further screening. We will exclude studies with any of the following conditions:

(1) duplicated publications;
(2) data are unavailable or incorrect, or no relevant data for meta-analysis (after at least 2 attempts to obtain essential additional information from the authors);
(3) meta-analysis of quasi randomized controlled clinical trials (defined as allocation using alternation, the sequence of admission, case record numbers, dates of birth), nonrandomized controlled clinical trials, or observational studies;
(4) systematic reviews without meta-analysis or meta-analyses without systematic reviews;
(5) meta-analysis of osteoarthritis in hand, hip, ankle, and other joints (if a meta-analysis evaluates a site other than the knees and presents the KOA results separately, it will be included);
(6) abstracts, commentaries, methodological studies, overviews, narrative reviews, and guidelines

### 2.5 Data extraction

All the retrieved studies will be imported into Zotero software (Corporation for Digital Scholarship, Vienna, Virginia 22182, USA), version 5.0.84^[28]^ and duplicates will be removed.

Initially, 2 reviewers (CAFA and LPO) independently will select the study abstracts. Consensus meetings will be held, and in case of disagreement, a third reviewer (IRG) will judge the abstract's relevance.

In the second stage, the same reviewers (CAFA and LPO) will read the full-text articles and decide about the inclusion or exclusion of them, also independently. Disagreements arising in the consensus meetings of the 2 reviewers (CAFA and LPO) will also be resolved with the same third reviewer (IRG).

A standardized data extraction form was designed. After identify all the included studies, the 2 reviewers will independently extract data, which including study characteristics (authors and published year), participant characteristics (sample size, age, sex, nationality, etc), methodological characteristics (interventions, comparisons, method of pooling and bias assessment, funding, and other relevant informations), results (number of studies included in the meta-analysis, risk of bias within included studies, number and percentage of studies included in the review according to quality, and other relevant informations), and funding sources.

### 2.6 Synthesis of included studies

A synthesis of the evidence will be conducted and data will be presented in tables. Aggregate participant data will be used.

We are planning to carry out a descriptive summary of the results of our systematic review. In the synthesis phase of the results of our systematic review, the 3 reviewers of the team will work together. In case of disagreements in this phase, the 3 reviewers will resolve through discussion. The inclusion criterion we defined allows us to accept articles (systematic reviews with meta-analysis) that use different comparators with hyaluronic acid and using different doses of hyaluronic acid, with patients with different degrees of knee joint involvement. We expect to find a high degree of heterogeneity.

Thus, we chose not to perform meta-analysis. In the final phase of the systematic review, we will describe and summarize key findings and conclusions of studies.

Subgroup analysis considering the influence of sex, age, outcomes, and type of comparators will be conducted

### 2.7 Methodological quality evaluation

Two reviewers (CAFA and LPO) will independently appraise the methodological quality of included meta-analyses using the Assessment of Multiple Systematic Reviews 2 (AMSTAR 2)^[29]^ tool. In case of disagreements, these 2 first reviewers will resolve through discussion or, if necessary, the third reviewer (IRG) will be involved.

The AMSTAR 2^[29]^ is a critical appraisal tool for systematic reviews that include randomized or non-randomized studies of healthcare interventions, or both. It consists of 16 items to assess 7 critical domains of systematic reviews. The 7 critical domains include:

i) protocol registered before the beginning of the review (item 2);
ii) adequacy of the literature search (item 4);
iii) justification for excluding individual studies (item 7);
iv) risk of bias (ROB) from individual studies included in the review (item 9);
v) appropriateness of meta-analytical methods (item 11);
vi) consideration of ROB when interpreting the results of the review (item 13);
vii) assessment of presence and likely impact of publication bias (item 15).

According to AMSTAR 2,^[29]^ we will classify the quality of included systematic reviews into 1 of the 4 levels of confidence: high, moderate, low, or critically low. High confidence refers to systematic reviews without non-critical weakness that provide comprehensive summaries of available studies. Moderate confidence refers to systematic reviews with >1 non-critical weakness. Low confidence refers to systematic reviews with at least 1 critical flaw. Critically low confidence refers to systematic reviews with >1 critical flaw.

## 3 Discussion

Although several guidelines^[13,15,16]^ recommend non-pharmacological measures such as first line therapy for KOA, we know that pain reduction is necessary to perform physical exercises.^[30]^ Furthermore, for better quality of life, it is also necessary to have adequate pain control at the beginning of diet therapy for weight loss.^[30]^

We would like to highlight that despite the fact that in the last 15 years many randomized controlled trials and at least 40 meta-analyzes have been published on the effectiveness and safety of IAHA for the treatment of KOA, there is still much controversy and uncertainty on this topic.^[31]^ If we consider only some of the meta-analyzes published in the past 5 years, we will see that some of them recommend IAHA as a very effective and safe treatment,^[24,32–34]^ while others contraindicate this intervention,^[31,35,36]^ both due to its low effectiveness, as well as the lack of safety. We can explain these conflicting results for several reasons: the low quality of the meta-analyzes,^[31]^ individual differences in the formulation of the IAHA,^[30]^ specific clinical characteristics of the patients^[37]^ and even the presence of conflicts of interest (declared or not) by the authors of the studies and/or financing of studies by the industry.^[38]^ The aspects of low methodological quality and potential conflicts of interest affecting the IAHA's effectiveness for KOA treatment report are important and should be better elucidated. Each of these situations was assessed in at least 1 meta-analysis.^[31,38]^ In the first meta-analysis,^[31]^ the authors assessed 31 systematic reviews about the efficacy and safety of HA for KOA therapy and assessed RoB using the ROBIS tool. There were only 41.9% of with low RoB (there were 13 systematic reviews with low RoB, 47 with high RoB, and 2 with unclear RoB). The second meta-analysis^[38]^ demonstrated that a potential conflict of interest was common. Industry funded 30 (63%) of the 48 studies assessing IAHA for KOA, and in 17 of these 30 industry-funded studies at least one of the authors was an employee of the sponsoring pharmaceutical company. This could be the reason for the more favorable conclusions regarding the efficacy IAHA for KOAitis. The absence of industry-authored studies with unfavorable conclusions about the efficacy of IAHA for KOA, raises concern that perhaps industry-sponsored trials with “unfavorable” results have not been published perhaps due to financial influence from the sponsoring industry.

It is important to highlight that this treatment is contraindicated in some situations like persons with known hypersensitivity to hyaluronate products, women who are pregnant or nursing, pediatric patients, patients with bacteremia, or patients with infections in or around the target knees.^[39]^ To avoid joint infection, intraarticular injections should always be performed under sterile conditions.^[39]^

We think that we will organize knowledge about this important topic of therapy of KOA with IAHA in a complete, organized, and comprehensive way by selecting only systematic reviews with meta-analysis of randomized clinical trials in a comprehensive and meticulous way and using AMSTAR 2 to assess the quality of selected meta-analyzes.

## Author contributions

**Conceptualization:** Carlos Augusto Ferreira De Andrade, Liszt Palmeira de Oliveira.

**Funding acquisition:** Max Rogério Freitas Ramos, Eduardo Costa Freitas da Silva, Liszt Palmeira de Oliveira.

**Investigation:** Carlos Augusto Ferreira De Andrade.

**Methodology:** Carlos Augusto Ferreira De Andrade.

**Visualization:** Sara Regina Neto Pereira, Liszt Palmeira de Oliveira.

**Writing – original draft:** Carlos Augusto Ferreira De Andrade, Isabel Ruguê Genov, Joao Mauricio Barreto, Max Rogério Freitas Ramos, Eduardo Costa Freitas da Silva, Liszt Palmeira de Oliveira.

**Writing – review & editing:** Carlos Augusto Ferreira De Andrade, Isabel Ruguê Genov, Sara Regina Neto Pereira, Joao Mauricio Barreto, Max Rogério Freitas Ramos, Eduardo Costa Freitas da Silva, Liszt Palmeira de Oliveira.
